# Association between Tonsillectomy and Cardiovascular Diseases in Adults

**DOI:** 10.3390/jpm14010016

**Published:** 2023-12-21

**Authors:** Sung Joon Park, Sei Young Lee, Hahn Jin Jung, Min Woo Park, Hyo Geun Choi, Heejin Kim, Jee Hye Wee

**Affiliations:** 1 Department of Otorhinolaryngology-Head and Neck Surgery, Chung-Ang University Gwangmyeong Hospital, Chung-Ang University College of Medicine, Gwangmyeong 14353, Republic of Korea; hypo23hns@cau.ac.kr; 2 Department of Otorhinolaryngology-Head and Neck Surgery, Chung-Ang University Hospital, Chung-Ang University College of Medicine, Seoul 06973, Republic of Korea; syleemd@cau.ac.kr; 3 Department of Otorhinolaryngology-Head and Neck Surgery, Chungbuk National University Hospital, Chungbuk National University College of Medicine, Cheongju 28644, Republic of Korea; hahnjin2@cbnu.ac.kr; 4 Department of Otorhinolaryngology-Head and Neck Surgery, Kangdong Sacred Heart Hospital, Seoul 05355, Republic of Korea; subintern@kdh.or.kr; 5 Department of Otorhinolaryngology-Head and Neck Surgery, Mdanalytics, Suseoseoulent Clinic, Seoul 06349, Republic of Korea; pupen@naver.com; 6 Department of Otorhinolaryngology-Head and Neck Surgery, Hallym University Sacred Heart Hospital, Hallym University College of Medicine, Anyang 14068, Republic of Korea; mir5020@hallym.or.kr

**Keywords:** tonsillectomy, cardiovascular disease, stroke, myocardial ischemia, obstructive sleep apnea

## Abstract

This study aimed to evaluate the association between tonsillectomy and cardiovascular diseases (CVDs) in the Korean adult population. Using data from the 2002–2015 Korean National Health Insurance Service-Health Screening Cohort, a total of 1082 participants aged 40 years or older who had undergone tonsillectomy were matched with 4328 control individuals for age, sex, income, and region of residence. We evaluated the incidence of CVDs in both the tonsillectomy and control groups and calculated the hazard ratios (HRs) of stroke, ischemic heart disease (IHD), and heart failure (HF) for participants who underwent tonsillectomy using a stratified Cox proportional hazard model. The incidence rates of stroke (81.3 vs. 46.6 per 10,000 person-years) and IHD (112.3 vs. 64.9 per 10,000 person-years) were significantly higher in patients who had undergone tonsillectomy than in the control group. After adjustment, the tonsillectomy group exhibited a 1.78-fold and 1.60-fold higher occurrence of stroke (CI = 1.32–2.42, *p* < 0.001) and IHD (CI = 1.24–2.08, *p* < 0.001), respectively, compared to the control group. However, there was no significant difference in the incidence rate of tonsillectomy and control groups (11.1 vs. 6.1 per 10,000 person-years). The HR of HF did not differ significantly between the tonsillectomy and control groups in the adjusted model (*p* = 0.513). We identified a significant relationship between a history of tonsillectomy and occurrence of stroke/IHD in the Korean adult population.

## 1. Introduction

Cardiovascular diseases (CVDs), particularly stroke and ischemic heart disease (IHD), stand as the primary causes of global disability and mortality. The death toll due to CVDs rose steadily from 12.1 million in 1990 to 18.6 million in 2019, constituting 32% of global deaths. The global trends for years of life lost and disability-adjusted life years have also seen a significant increase, with years lived with disability doubling from 17.7 million in 1990 to 34.4 million in 2019 [1]. According to the Korea Heart Disease Fact Sheet 2020, published by the Korean Society of Cardiology, the CVD mortality rate declined until 2010, subsequently experiencing a steady increase to 123 per 100,000 persons in 2018 [2]. The global burden of CVD has persistently risen over the last two decades, prompting increased global attention to CVD-related risk factors.

Tonsillectomy stands out as one of the most prevalent surgeries performed in otorhinolaryngology and head and neck surgery departments. In 2006, the Centers for Disease Control and Prevention reported that out of 737,000 total tonsillectomies in the United States, 297,000 were performed on patients aged 15 years or older [3]. According to a 35-year epidemiological study, the overall number of tonsillectomies has increased, and indications for surgery have shifted from infection to upper airway obstruction from 1970 to 2005 [4]. In addition, previous studies have shown that tonsillectomy can have systemic impacts, including immunoglobulin A nephropathy [5], appendicitis [6], inflammatory bowel disease [7], periodontitis [8], and cancers [9].

Several studies have reported the impact of adenotonsillectomy on cardiovascular parameters in children with obstructive sleep apnea (OSA) [10,11], although the results remain controversial. Because OSA in children is primarily caused by tonsil and adenoid hypertrophy, adenotonsillectomy is considered the first-line treatment for pediatric OSA. A systematic review noted improvements in cardiovascular parameters, including blood pressure (BP), heart rate, and cardiac morphology and function, following adenotonsillectomy in children with OSA [10]. In contrast, another meta-analysis revealed no significant decreases in office systolic BP, diastolic BP, or 24 h ambulatory BP after adenotonsillectomy in children with OSA [11]. Furthermore, a prospective Swedish cohort study reported that tonsillectomy performed prior to the age of 20 years was associated with an increased risk for acute myocardial infarction (AMI) later in life, while tonsillectomy performed at or above 20 years of age did not show any association [12].

In contrast to research focusing on children, there are few studies addressing the correlation between CVDs and tonsillectomy, despite the fact that OSA is well known to be associated with CVDs in adults [13,14]. Given the shift in indications for tonsillectomy toward upper airway obstruction, our hypothesis posited that adult patients aged 40 and above requiring tonsillectomy were likely to have experienced chronic upper airway obstruction for a long period of time, resulting in an increased risk of CVDs even if they underwent tonsillectomy. To verify this hypothesis, we utilized a national cohort database to evaluate the risk of CVDs in Korean adults who underwent tonsillectomy compared to controls.

## 2. Materials and Methods

### 2.1. Ethics

The present study received approval from the Hallym University Ethics Committee (2019-10-023). Due to the impracticalities and minimal risk associated with the retrospective design of the study, the Institutional Review Board waived the requirement for written informed consent. All analyses adhered to the guidelines and regulations set forth by the Hallym University Ethics Committee.

### 2.2. Participant Selection

A case-control study utilized the data from the Korean National Health Insurance Service-Health Screening Cohort (NHIS-HEALS). A comprehensive overview of the NHIS-HEALS data is available in a previous study [15].

**Figure 1:**
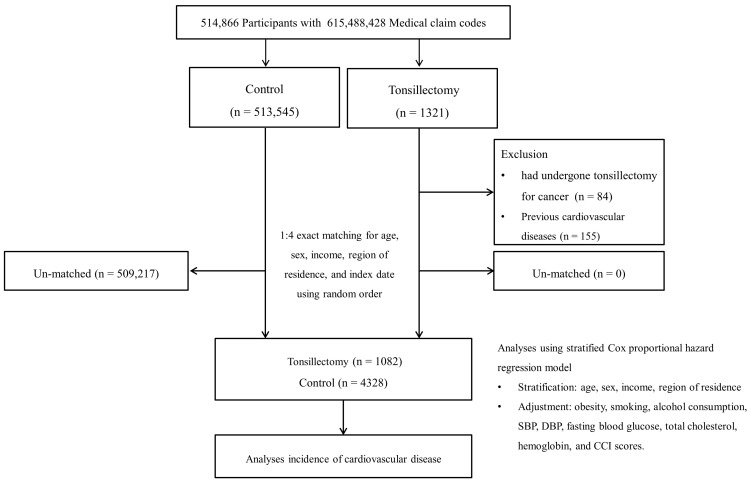
Schematic illustration of the participant selection process used in the present study.

Participants who underwent tonsillectomy were selected from 514,866 participants with 615,488,428 medical claim codes recorded between 2002 and 2015 (*n* = 1321). Controls were defined as those who did not undergo tonsillectomy during the same period (*n* = 513,545). Tonsillectomy participants were excluded if the surgery was performed for cancer (*n* = 84). Tonsillectomy participants were 1:4 matched with controls based on age, sex, income, and region of residence. To prevent selection bias in matching, the controls were randomly sorted and selected from top to bottom. It was assumed that the matched control participants were evaluated simultaneously with each matched tonsillectomy participant (index date). Consequently, some control participants died before the index date were excluded. Both tonsillectomy and control groups excluded participants with a history of stroke, IHD, or HF before the index date. As a result, 155 participants with a history of CVDs before the index date were excluded. During the matching procedure, 509,217 control participants were excluded. Ultimately, 1082 tonsillectomy participants were 1:4 matched with 4328 control participants (Figure 1).

### 2.3. Definition of Tonsillectomy (Independent Variable)

Tonsillectomy was defined using claim codes Q2300 [16]. Among participants who had undergone tonsillectomy, those who had it for cancer were excluded (*n* = 84).

### 2.4. Definition of Cardiovascular Diseases

Patients with stroke, IHD, and HF were identified using ICD-10 codes (I60–I69 for stroke, I20–I25 for IHD, and I50 for HF). For each disease, only participants hospitalized for ≥2 days or who died from the disease were included, as described in our previous studies [17,18]. In addition, patients with hemorrhagic stroke (I60–I62) and ischemic stroke (I63) were included.

### 2.5. Covariates

Age groups were segmented into 5-year intervals, resulting in a total of 10 age groups. Income categories were delineated into 5 levels (level 1 [lowest income]–5 [highest income]). The area of residence was classified into urban and rural areas. Smoking and alcohol consumption status, obesity assessed by body mass index (BMI, kg/m^2^), and levels of systolic BP (SBP, mmHg), diastolic BP (DBP, mmHg), fasting blood glucose (mg/dL), total cholesterol (mg/dL), and hemoglobin (g/dL) were determined [19]. The Charlson Comorbidity Index (CCI) was measured as a continuous variable (ranging from 0 [no comorbidities] to 29 [multiple comorbidities]) [20,21], excluding cerebrovascular diseases, AMI, or congestive heart failure.

### 2.6. Statistical Analyses

To compare the general characteristics between the tonsillectomy and control groups, the absolute standardized difference (SD) was employed. An absolute SD below 0.20 was deemed a sign of achieving satisfactory balance [22].

Stratified Cox proportional hazard models were utilized to assess the hazard ratios (HRs) and 95% confidence intervals (CIs) of tonsillectomy for CVD. In this analysis, both crude (simple) and adjusted models (factoring in obesity, smoking status, alcohol consumption, SBP, DBP, fasting blood glucose, total cholesterol, and hemoglobin levels, and CCI scores) were employed. The analysis was stratified by matching variables, including age, sex, income, and region of residence. Kaplan–Meier curves were generated, and log-rank tests were conducted.

For subgroup analyses using the stratified Cox proportional hazards model, participants were divided by age (<50 years old and ≥50 years old), sex (male and female), income (low and high), and region of residence (urban and rural).

Two-tailed analyses were conducted, with significance defined as a *p*-value less than 0.05. Statistical analyses were performed using SAS version 9.4 (SAS Institute Inc., Cary, NC, USA).

## 3. Results

Table 1 shows the general characteristics of the participants. Matched participants did not differ in terms of age, sex, income, and region of residence (all SD = 0.00). The distributions of obesity, smoking status, alcohol consumption, SBP, DBP, fasting blood glucose, total cholesterol, and hemoglobin levels, and CCI scores exhibited no significant differences between the tonsillectomy and control groups.

### 3.1. The Incidence of Stroke

**Figure 2:**
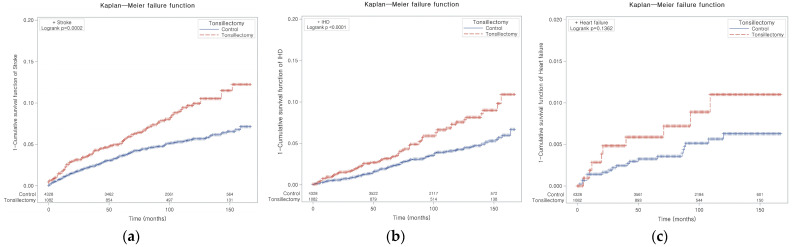
Kaplan–Meier curves of (**a**) stroke, (**b**) ischemic heart disease, and (**c**) heart failure in patients who underwent tonsillectomy.

Stroke occurred in 5.9% of the patients who underwent tonsillectomy and 3.4% of the control participants (Table 1). In the tonsillectomy group, the stroke incidence rates were 81.9 per 10,000 person-years, while in the control group, the rate was 46.6 per 10,000 person-years (Table 2). The Kaplan–Meier analysis and log-rank test demonstrated an increased risk of developing stroke in patients who had undergone tonsillectomy in comparison to the control participants (*p* = 0.0002, Figure 2a).

A higher HR of stroke for patients who underwent tonsillectomy was found in both crude (HR = 1.75, CI = 1.30–2.35, *p* < 0.001) and adjusted (HR = 1.78, CI = 1.32–2.42, *p* < 0.001) models (Table 2). In subgroup analyses based on age and sex, the incidence of stroke was significantly higher among older patients (≥50 years old) (HR = 2.10, CI = 1.43–3.09, *p* < 0.001) and male patients (HR = 1.88, CI = 1.31–2.71, *p* = 0.001) in the adjusted model (Table 2). All income and region of residence subgroups displayed a higher HR of stroke for patients in the tonsillectomy group compared to those in the control group.

### 3.2. The Incidence of IHD

IHD occurred in 8.0% of the patients in the tonsillectomy group and 4.7% of the patients in the control group (Table 1). The incidence rates for IHD were 112.3 and 64.9 per 10,000 person-years in the tonsillectomy and control groups, respectively (Table 3). As depicted in Figure 2b, the occurrence of IHD was higher in patients who had undergone tonsillectomy than in those who had not undergone tonsillectomy (*p* < 0.0001).

The Cox proportional hazard model indicated an elevated likelihood of developing IHD in patients who underwent tonsillectomy compared to those who did not, even after adjusting for factors such as obesity, smoking status, alcohol consumption, SBP, DBP, fasting blood glucose, total cholesterol, and hemoglobin levels, and CCI scores (HR = 1.60, CI = 1.24–2.08, *p* < 0.001, Table 3). Subgroup analyses for participants aged <50 years old (HR = 2.02, CI = 1.34–3.05, *p* = 0.001), men (HR = 1.57, CI = 1.15–2.14, *p* = 0.004) and women (HR = 1.66, CI = 1.01–2.72, *p* = 0.044), participants with high income (HR = 2.03, CI = 1.42–2.89, *p* < 0.001), and urban residents (HR = 2.08, CI = 1.43–3.02, *p* < 0.001) revealed a significant association of tonsillectomy with IHD in the adjusted model (Table 3).

### 3.3. The Incidence of HF

In the tonsillectomy group, 0.8% of patients developed HF compared to 0.5% in the control group (Table 1). The incidence rate of HF showed no significant difference between tonsillectomy and control groups (11.1 vs. 6.1 per 10,000 person-years, Table 4; *p* = 0.1362, log-rank test, Figure 2c). Subgroup analysis stratified by age, sex, income, and region of residence indicated that the HR of HF was not significantly different between the tonsillectomy and control groups in the adjusted model (all *p* > 0.05, Table 4).

## 4. Discussion

We observed that a history of tonsillectomy in adults correlated with a higher incidence of stroke and IHD but not with HF. In the subgroup analyses, the elevated risk of stroke in the tonsillectomy group was more pronounced among participants in the older age group (≥50 years old) and males, irrespective of income and region of residence. In addition, the increased risk of IHD in the tonsillectomy group was more significant among participants in the younger age group (<50 years old), those with higher income, and those residing in urban areas, regardless of sex.

Although the causality cannot be determined based on these results, some eligible explanations are possible for the present findings. Firstly, tonsillectomy in adulthood, especially after the age of 40 years, may not be helpful in reversing the risk of CVDs due to exposure to long-term chronic upper airway obstruction. As highlighted in a prior epidemiological study, the indication of tonsillectomy has shifted from infection to upper airway obstruction [4]. Consequently, tonsillectomy has become a valuable treatment modality for OSA in both children and adults. A systematic review underscored the significant role of adenotonsillectomy in children in reversing the cardiovascular consequences of OSA [10]. Furthermore, a meta-analysis indicated that isolated tonsillectomy can be efficacious as a treatment for selected adult OSA patients (those with large tonsils and an apnea hypopnea index < 30/h) [23]. Nevertheless, the applicability of previous findings on tonsillectomy for adult OSA patients is constrained. For instance, a previous population-based study in Korea reported that tonsillectomy performed in adults aged over 40 years does not result in changes in weight or BP [24]. Our study’s results further revealed that adult patients undergoing tonsillectomy faced an increased risk of stroke and IHD. Furthermore, if tonsillectomy is performed later in life, especially in individuals over 50 years old, it significantly amplifies the risk of stroke. We hypothesized that the duration of OSA might influence the reversibility of its impact on stroke risk. Consequently, we recommend suggesting tonsillectomy for adult patients with OSA before the age of 50 years to mitigate the heightened risk of stroke. Nevertheless, additional studies are necessary to validate our hypothesis and ascertain potential underlying mechanisms for sex differences associated with tonsillectomy in adulthood and the risk of stroke.

Secondly, changes in immune function following tonsillectomy may potentially contribute an elevated risk of CVDs. The impact of tonsillectomy on immune function has been a subject of controversy, and its effects may differ between children and adults. A prior meta-analysis indicated that tonsillectomy has no clinically significant adverse effects on the immune system, encompassing both humoral and cellular immunity [25]. In contrast, a previous Korean population-based study reported that tonsillectomy increases the risk of deep neck infection in adults but not in children [26]. They suggested that the varying compensation for immunological function between children and adults could influence the heightened risk of deep neck infection in adults. Moreover, a prior population-based study in Sweden demonstrated that tonsillectomy before adulthood is linked to an increased risk for AMI [12]. In that study, they also suggested the possibility that alterations in immune function mediates the effects of the tonsillectomy on the heightened AMI risk. The findings of the present study also revealed that tonsillectomy in adulthood was associated with a higher incidence of not only stroke but also IHD. Atherosclerosis, a well-known pathophysiological mechanism for IHD, is considered a form of inflammatory process. Several chronic inflammatory disorders and autoimmune diseases have been reported to have a positive association with the risk for IHD [27,28]. In addition, it has been reported that C-reactive protein levels and other markers of systemic inflammation can anticipate the risk of IHD nearly as effectively as other traditional risk factors [29,30]. Based on the firm relationship between inflammation and IHD, the surgical removal of tonsils, which are secondary lymphoid organs and part of the mucosa-associated lymphoid tissue system, may impact the immunological status of a patient, leading to an increased risk of IHD. However, it is crucial not to interpret the findings as conclusive evidence that tonsillectomy causes CVDs.

Thirdly, the mechanism behind the relationship between tonsillectomy and the risk of CVDs has not been definitively established but is believed to involve the intricate interplay of chronic inflammation and host immunity. Similar to infectious diseases, we speculate that a reduced probability of compensating for the diminished immunological function may offer a credible rationale for the heightened risk of chronic inflammation and CVDs in the adult group in this study. While there is a counterargument suggesting that the removal of the tonsils can decreased the amount of bacteria in the upper respiratory tract, leading to reduced inflammation and improved cardiovascular health, a Cochrane database of systematic reviews has determined that available evidence is insufficient to establish definite conclusions regarding the efficacy of tonsillectomy in adults experiencing chronic and recurrent acute tonsillitis [31].

Furthermore, mounting evidence suggests that microbiota play a crucial role in maintaining cardiovascular health and their dysregulation may contribute to the development of CVDs [32,33]. Notably, oral dysbiosis, an inflammation-associated molecular and cellular mechanism, is increasingly being linked to CVDs [34,35]. The absence of tonsils might induce dysbiosis of the oral microbiota, which may contribute to increased CVD risk. A previous population-based study in Taiwan reported a 1.84-fold higher risk of irritable bowel syndrome (IBS) in tonsillectomy patients, with a higher risk observed in patients aged ≥50 years old [36]. They suspected that tonsillectomy might exacerbate dysbiosis of the gut microbiome, increasing the risk of IBS, which may be a similar plausible mechanism to our hypothesis.

Contrary to the findings of our study, a recent European cohort study involving over 20,000 patients reported a lower prevalence of cardiovascular comorbidities (47.3% vs. 56.5%) in patients who had undergone tonsillectomy [37]. However, the results did not reach statistical significance, and relevant confounders, such as obesity, which affect CVDs, were not corrected for. Another prospective multi-institutional study in United States suggested a beneficial effect of adult tonsillectomy alone in OSA management [38], but the study only included young adults with an average age of 27.9 years. To clarify whether the long-term OSA actually affect the incidence of CVDs, a further prospective study is needed in participants underwent tonsillectomy in childhood, young adults, and older adults.

Several limitations must be acknowledged in the present study. Firstly, being a retrospective observational cohort study, causality cannot be established and inherent bias may be present. Given these limitations, further prospective longitudinal research, including polysomnographic data for the diagnosis of OSA and more detailed information on the indication of tonsillectomy, is essential to validate the current findings and establish causation. Secondly, it was unknown whether the participants undergoing tonsillectomy had OSA or chronic tonsillitis. The underlying etiology of tonsillectomy may have influenced the incidence of CVDs, and the lack of detailed explanations is a limitation. However, because OSA is highly prevalent in obese patients [39,40] and 76.6% of participants in this study were overweight or obese, it can be assumed that they were more likely to have underlying OSA. Thirdly, unlike the association between tonsillectomy and the risk of stroke and IHD, tonsillectomy history was not related to the incidence of HF. This is presumably due to the relatively low incidence of HF (<1%) compared to stroke and IHD. Lastly, it cannot be generalized about these association in other races than Koreans, considering the specific demographic and health system context of the Korean. Despite these limitations, the study’s strength lies in its population-based nature, involving numerous participants and an extended follow-up duration. No participants were lost during the follow-up, and there is no possibility of recall bias since the Korean National Health Insurance system covers almost the entire population in Korea. Furthermore, we adjusted for several confounding factors, including comorbidity variables (such as fasting blood glucose, cholesterol, and hemoglobin levels and CCI scores) and lifestyle factors (status of smoking and alcohol consumption).

## 5. Conclusions

Tonsillectomy experienced in adult populations showed a significant association with incidence of stroke and IHD. We may suggest that tonsillectomy in adulthood, especially after the age of 40 years, may not be helpful in reversing the risk of CVDs due to exposure to long-term chronic upper airway obstruction. Further comprehensive prospective studies are warranted to unravel the biological mechanisms underpinning these associations. Nonetheless, physicians should be vigilant regarding the potential risk of developing CVDs in patients undergoing tonsillectomy in adulthood.

## Figures and Tables

**Table 1:** General characteristics of participants.

Characteristics	Total Participants		
	Tonsillectomy	Control	Standardized Difference
Total number (*n*, %)	1082 (100.0)	4328 (100.0)	
Age (years old) (*n*, %)			0.00
40–44	166 (15.3)	664 (15.3)	
45–49	287 (26.5)	1148 (26.5)	
50–54	293 (27.1)	1172 (27.1)	
55–59	207 (19.1)	828 (19.1)	
60–64	84 (7.8)	336 (7.8)	
65–69	35 (3.2)	140 (3.2)	
70–74	7 (0.7)	28 (0.7)	
75–79	2 (0.2)	8 (0.2)	
80–84	1 (0.1)	4 (0.1)	
85+	79 (1.0)	316 (1.0)	
Sex (*n*, %)			0.00
Male	755 (69.8)	3020 (69.8)	
Female	327 (30.2)	1308 (30.2)	
Income (*n*, %)			0.00
1 (lowest)	98 (9.1)	392 (9.1)	
2	119 (11.0)	476 (11.0)	
3	132 (12.2)	528 (12.2)	
4	223 (20.6)	892 (20.6)	
5 (highest)	510 (47.1)	2040 (47.1)	
Region of residence (*n*, %)			0.00
Urban	526 (48.6)	2104 (48.6)	
Rural	556 (51.4)	2224 (51.4)	
Obesity (*n*, %) *			0.33
Underweight	5 (0.5)	66 (1.5)	
Normal	248 (22.9)	1470 (34.0)	
Overweight	295 (27.3)	1229 (28.4)	
Obese I	471 (43.5)	1443 (33.3)	
Obese II	63 (5.8)	120 (2.8)	
Smoking status (*n*, %)			0.1
Nonsmoker	634 (58.6)	2548 (58.6)	
Past smoker	189 (17.5)	623 (17.5)	
Current smoker	259 (23.9)	1157 (23.9)	
Alcohol consumption (*n*, %)			0.02
<1 time a week	649 (60.0)	2555 (59.0)	
≥1 time a week	433 (40.0)	1773 (41.0)	
Systolic blood pressure (*n*, %)			0.04
<120 mmHg	336 (31.1)	1425 (32.9)	
120–139 mmHg	544 (50.3)	2123 (49.1)	
≥140 mmHg	202 (18.7)	780 (18.0)	
Diastolic blood pressure (*n*, %)			0.05
<80 mmHg	456 (42.1)	1931 (44.6)	
80–89 mmHg	403 (37.3)	1577 (36.4)	
≥90 mmHg	223 (20.6)	820 (19.0)	
Fasting blood glucose (*n*, %)			0.08
<100 mg/dL	694 (64.1)	2898 (67.0)	
100–125 mg/dL	313 (28.9)	1108 (25.6)	
≥126 mg/dL	75 (6.9)	322 (7.4)	
Total cholesterol (*n*, %)			0.05
<200 mg/dL	558 (51.6)	2271 (52.5)	
200–239 mg/dL	363 (33.5)	1489 (34.4)	
≥240 mg/dL	161 (14.9)	568 (13.1)	
Hemoglobin (g/dL)			0.09
≥12 for men and ≥10 for women	1077 (99.5)	4274 (98.8)	
<12 for men and <10 for women	5 (0.5)	54 (1.3)	
CCI score ^†^			0.21
0	786 (72.6)	3466 (80.1)	
1	186 (17.2)	439 (10.1)	
≥2	110 (10.2)	423 (9.8)	
Stroke	64 (5.9)	149 (3.4)	0.12
Hemorrhagic stroke	3 (0.3)	16 (0.4)	0.02
Ischemic stroke	26 (2.4)	59 (1.4)	0.08
Other stroke	35 (3.2)	74 (1.7)	0.1
Ischemic heart disease	86 (8.0)	204 (4.7)	0.13
Heart failure	9 (0.8)	20 (0.5)	0.05

* Obesity (BMI, body mass index, kg/m^2^) was categorized as <18.5 (underweight), ≥18.5 to <23 (normal), ≥23 to <25 (overweight), ≥25 to <30 (obese I), and ≥30 (obese II). ^†^ CCI scores were calculated without cerebrovascular disease, acute myocardial infarction, and congestive heart failure.

**Table 2:** Crude and adjusted hazard ratios (95% confidence interval) of tonsillectomy compared to control group for stroke with stratified subgroup according to age, sex, income, and region of residence.

Independent Variables	Stroke/Participants (*n*, %)	Follow-Up Duration (PY)	IR per 10,000 (PY)	Hazard Ratios (95% CI) for Stroke			
				Crude ^†^	*p*-Value	Adjusted ^† ‡^	*p*-Value
Total participants (*n* = 5410)							
Tonsillectomy	64/1082 (5.9)	7876	81.3	1.75 (1.30–2.35)	<0.001 *	1.78 (1.32–2.42)	<0.001 *
Control	149/4328 (3.4)	31,966	46.6	1		1	
Age < 50 years old (*n* = 2265)							
Tonsillectomy	22/453 (4.9)	4386	50.2	1.38 (0.85–2.24)	0.196	1.42 (0.86–2.34)	0.176
Control	64/1812 (3.5)	17,644	36.3	1		1	
Age ≥ 50 years old (*n* = 3145)							
Tonsillectomy	42/629 (6.7)	3490	120.3	2.04 (1.41–2.96)	<0.001 *	2.10 (1.43–3.09)	<0.001 *
Control	85/2516 (3.4)	14,322	59.3	1		1	
Men (*n* = 3775)							
Tonsillectomy	45/755 (6.0)	5532	81.3	1.79 (1.26–2.55)	0.001 *	1.88 (1.31–2.71)	0.001 *
Control	103/3020 (3.4)	22,473	45.8	1		1	
Women (*n* = 1635)							
Tonsillectomy	19/327 (5.8)	2344	81.1	1.66 (0.97–2.83)	0.065	1.54 (0.87–2.70)	0.137
Control	46/1308 (3.5)	9493	48.5	1		1	
High income (*n* = 2860)							
Tonsillectomy	37/572 (6.5)	4180	88.5	1.87 (1.27–2.77)	0.002 *	1.95 (1.30–2.94)	0.001 *
Control	79/2288 (3.5)	16,968	46.6	1		1	
Low income (*n* = 2550)							
Tonsillectomy	27/510 (5.3)	3696	73.1	1.61 (1.03–2.51)	0.037 *	1.61 (1.02–2.55)	0.041 *
Control	70/2040 (3.4)	14,998	46.7	1		1	
Urban residents (*n* = 2630)							
Tonsillectomy	29/526 (5.5)	3776	76.8	2.14 (1.37–3.37)	0.001 *	2.30 (1.44–3.69)	0.001 *
Control	55/2104 (2.6)	15,382	35.8	1		1	
Rural residents (*n* = 2780)							
Tonsillectomy	35/556 (6.3)	4100	85.4	1.52 (1.03–2.24)	0.036 *	1.56 (1.04–2.33)	0.033 *
Control	94/2224 (4.2)	16,584	56.7	1		1	

* Stratified Cox proportional hazard regression model, Significance at *p* < 0.05. ^†^ Models were stratified by age, sex, income, and region of residence. ^‡^ The model was adjusted for obesity, smoking, alcohol consumption, systolic blood pressure, diastolic blood pressure, fasting blood glucose, total cholesterol, hemoglobin, and CCI scores.

**Table 3:** Crude and adjusted hazard ratios (95% confidence interval) of tonsillectomy compared to control group for ischemic heart disease (IHD) with stratified subgroup according to age, sex, income, and region of residence.

Independent Variables	IHD/Participants (*n*, %)	Follow-Up Duration (PY)	IR per 10,000 (PY)	Hazard Ratios (95% CI) for IHD			
				Crude ^†^	*p*-Value	Adjusted ^† ‡^	*p*-Value
Total participants (*n* = 5410)							
Tonsillectomy	86/1082 (7.9)	7661	112.3	1.73 (1.34–2.23)	<0.001 *	1.60 (1.24–2.08)	<0.001 *
Control	204/4328 (4.7)	31,446	64.9	1		1	
Age < 50 years old (*n* = 2265)							
Tonsillectomy	37/453 (8.2)	4249	87.1	2.19 (1.47–3.27)	<0.001 *	2.02 (1.34–3.05)	0.001 *
Control	68/1812 (3.8)	17,509	38.8	1		1	
Age ≥ 50 years old (*n* = 3145)							
Tonsillectomy	49/629 (7.8)	3412	143.6	1.49 (1.07–2.07)	0.017 *	1.39 (0.99–1.96)	0.057
Control	136/2516 (5.4)	13,937	97.6	1		1	
Men (*n* = 3775)							
Tonsillectomy	62/755 (8.2)	5396	114.9	1.76 (1.30–2.37)	<0.001 *	1.57 (1.15–2.14)	0.004 *
Control	144/3020 (4.8)	22,131	65.1	1		1	
Women (*n* = 1635)							
Tonsillectomy	24/327 (7.3)	2265	106.0	1.66 (1.03–2.67)	0.038 *	1.66 (1.01–2.72)	0.044 *
Control	60/1308 (4.6)	9315	64.4	1		1	
High income (*n* = 2860)							
Tonsillectomy	51/572 (8.9)	4040	126.2	2.14 (1.52–3.00)	<0.001 *	2.03 (1.42–2.89)	<0.001 *
Control	99/2288 (4.3)	16,755	59.1	1		1	
Low income (*n* = 2550)							
Tonsillectomy	35/510 (6.9)	3621	96.7	1.36 (0.92–1.99)	0.120	1.28 (0.87–1.89)	0.218
Control	105/2040 (5.1)	14,691	71.5	1		1	
Urban residents (*n* = 2630)							
Tonsillectomy	45/526 (8.6)	3680	122.3	2.07 (1.45–2.97)	<0.001 *	2.08 (1.43–3.02)	<0.001 *
Control	89/2104 (4.2)	15,103	58.9	1		1	
Rural residents (*n* = 2780)							
Tonsillectomy	41/556 (7.4)	3981	103.0	1.46 (1.02–2.09)	0.037 *	1.31 (0.91–1.88)	0.154
Control	115/2224 (5.2)	16,343	70.4	1		1	

* Stratified Cox proportional hazard regression model, Significance at *p* < 0.05. ^†^ Models were stratified by age, sex, income, and region of residence. ^‡^ The model was adjusted for obesity, smoking, alcohol consumption, systolic blood pressure, diastolic blood pressure, fasting blood glucose, total cholesterol, hemoglobin, and CCI scores.

**Table 4:** Crude and adjusted hazard ratios (95% confidence interval) of tonsillectomy compared to control group for heart failure with stratified subgroup according to age, sex, income, and region of residence.

Independent Variables	Heart Failure/Participants (*n*, %)	Follow-Up Duration (PY)	IR per 10,000 (PY)	Hazard Ratios (95% CI) for Heart Failure			
				Crude ^†^	*p*-Value	Adjusted ^† ‡^	*p*-Value
Total participants (*n* = 5410)							
Tonsillectomy	9/1082 (0.8)	8121	11.1	1.79 (0.81–3.93)	0.148	1.35 (0.55–3.27)	0.513
Control	20/4328 (0.5)	32,556	6.1	1		1	
Age < 50 years old (*n* = 2265)							
Tonsillectomy	3/453 (0.7)	4458	6.7	2.00 (0.50–8.01)	0.326	2.79 (0.63–12.39)	0.177
Control	6/1812 (0.3)	17,909	3.4	1		1	
Age ≥ 50 years old (*n* = 3145)							
Tonsillectomy	6/629 (1.0)	3663	16.4	1.70 (0.65–4.42)	0.279	3.25 (0.96–11.04)	0.059
Control	14/2516 (0.6)	14,647	9.6	1		1	
Men (*n* = 3775)							
Tonsillectomy	7/755 (0.9)	5702	12.3	3.51 (1.27–9.67)	0.015 *	1.70 (0.65–4.42)	0.278
Control	8/3020 (0.3)	22,910	3.5	1		1	
Women (*n* = 1635)							
Tonsillectomy	2/327 (0.6)	2419	8.3	0.66 (0.15–2.93)	0.580	0.74 (0.13–4.25)	0.732
Control	12/1308 (0.9)	9646	12.4	1		1	
High income (*n* = 2860)							
Tonsillectomy	6/572 (1.0)	4309	13.9	1.70 (0.65–4.42)	0.278	1.38 (0.47–4.07)	0.555
Control	14/2288 (0.6)	17,255	8.1	1		1	
Low income (*n* = 2550)							
Tonsillectomy	3/510 (0.6)	3812	7.9	2.00 (0.50–7.99)	0.328	2.23 (0.39–12.87)	0.369
Control	6/2040 (0.3)	15,301	3.9	1		1	
Urbal residents (*n* = 2630)							
Tonsillectomy	4/526 (0.8)	3911	10.2	5.16 (1.16–23.07)	0.032 *	1.18 (0.43–3.20)	0.748
Control	3/2104 (0.1)	15,613	1.9	1		1	
Rural residents (*n* = 2780)							
Tonsillectomy	5/556 (0.9)	4210	11.9	7.31 (1.05–50.94)	0.045 *	0.81 (0.24–2.73)	0.734
Control	17/2224 (0.8)	16,943	10.0	1		1	

* Stratified Cox proportional hazard regression model, Significance at *p* < 0.05. ^†^ Models were stratified by age, sex, income, and region of residence. ^‡^ The model was adjusted for obesity, smoking, alcohol consumption, systolic blood pressure, diastolic blood pressure, fasting blood glucose, total cholesterol, hemoglobin, and CCI scores.

## Data Availability

Data in this study were from the Korean National Health Insurance Service-Health Screening Cohort. Release of the data by the researcher is not legally allowed. All data are available from the database of the National Health Insurance Sharing Service (NHISS) (https://nhiss.nhis.or.kr/ (accessed on 10 January 2022)). The NHISS allows all data for any researcher who promises to follow the research ethics at some cost. If you want to access the data of this article, you can download it from the website after promising to follow the research ethics.
